# Vaginal Infections— A User-Friendly Taxonomy

**DOI:** 10.1155/S1064744995000469

**Published:** 1995

**Authors:** J. Martinez de Oliveira

**Affiliations:** University of Porto School of Medicine Porto Portugal


Infectious Diseases in Obstetrics and Gynecology 3:133–134 (1995)
© 1995 Wiley-Liss, Inc.

### Vaginal Infections—A User-Friendly Taxonomy

English has become the internationally shared language for science. Being the main language used in meetings and publications, English must be translated to the various languages of the international scientific community. The material to be translated includes every text presenting a conclusion, consensus, or "law" in every field of knowledge. Therefore, the use of specific terms is mandatory in preventing the emergence of conflicting ideas and definitions both in the original English version and in its translation.

The terms *vaginal infection, vaginitis,* and *vaginal discharge* are frequently but erroneously used as synonymous, particularly in the English literature.

*Vaginal infection* is an objective disturbance of the vaginal fluids or vaginal walls resulting from either an abnormal increase in the normal (indigenous), but potentially pathogenic microbiologic constituents of the vagina or from an increase in pathogenic microorganisms that normally do not inhabit the vagina. This definition is both qualitative with respect to the abnormal vaginal inhabitants such as *Trichomonas vaginalis* and quantitative with respect to the normal vaginal microorganisms such as the anaerobic flora. Clinically, a vaginal infection presents itself as a vaginitis, a vaginal discharge, or both.

*Vaginitis* is defined as the presence of an inflammatory reaction in the walls of the vagina and, by extension, the malpighian-covered ectocervix. Although the most frequent cause of vaginitis is a vaginal infection, there are non-infectious causes of vaginitis, an example being contact vaginitis caused by antiseptics or sperm.

With this definition of vaginitis, the diagnosis is based on histologic, macroscopic, or colposcopic findings. Clearly, the presence of inflammatory cells in the vaginal fluid is not diagnostic of vaginitis, as these cells may have their origin elsewhere, for example, in the cervix or endometrium.

*Vaginal discharge* has two meanings. Subjectively, a vaginal discharge is the patient's perception of a disturbing increase in the moisture of the external genitalia, usually, but not always, the consequence of an increased amount of fluid draining from or through the vagina. As a clinical sign, a vaginal discharge is represented by an increased amount of fluid in the vagina objectively observed as a large pool by the clinician during a speculum examination. By extension, discharges from the cervix, endometrium, or fallopian tubes are referred to as "vaginal" discharges because they pass through the vagina.

Even though a vaginal discharge as a symptom is commonly related to an objective vaginal discharge, this relationship is not always proven. A divergence results from the sensitivity of individual patients, with some women suffering from only small amounts of fluid flowing onto the vulva and others without complaints who are found during a speculum examination to harbor enormous amounts of fluid in the vagina.

A clinically proven vaginal discharge may have an non-infectious or, not uncommonly, a physiologic etiology. During sexual excitement, the peri-ovulatory phase of the menstrual cycle, and pregnancy, a "normal" vaginal discharge is

Received November 21, 1995
Accepted November 21, 1995

Editorial usually experienced and clinically evident. For these physiologic etiologies, there is no need for treatment beyond a simple explanation and reassurance to the patient.

The difficulty and confusion in interpreting these terms not only in English but in translating them into other languages exemplify our need for a nomenclature based on commonly accepted meanings of medical terms. Having such a nomenclature would benefit us by improving our scientific communication and clinical practices.

*J. Martinez de Oliveira, M.D., Ph.D.*
*Associate Professor of Gynecology*
*University of Porto School of Medicine*
*Porto, Portugal*

